# PASS: A Multimodal Database of Physical Activity and Stress for Mobile Passive Body/ Brain-Computer Interface Research

**DOI:** 10.3389/fnins.2020.542934

**Published:** 2020-12-08

**Authors:** Mark Parent, Isabela Albuquerque, Abhishek Tiwari, Raymundo Cassani, Jean-François Gagnon, Daniel Lafond, Sébastien Tremblay, Tiago H. Falk

**Affiliations:** ^1^INRS-EMT, Université du Québec, Montréal, QC, Canada; ^2^Thales Research and Technology Canada, Quebec City, QC, Canada; ^3^Université Laval, School of Psychology, Quebec City, QC, Canada; ^4^PERFORM Center, Concordia University, Montréal, QC, Canada

**Keywords:** neurophysiology, wearables, physical activity, stress, body/brain-computer interfaces, heart rate variability, electroencephalography (EEG), electrodermal activity

## Abstract

With the burgeoning of wearable devices and passive body/brain-computer interfaces (B/BCIs), automated stress monitoring in everyday settings has gained significant attention recently, with applications ranging from serious games to clinical monitoring. With mobile users, however, challenges arise due to other overlapping (and potentially confounding) physiological responses (e.g., due to physical activity) that may mask the effects of stress, as well as movement artifacts that can be introduced in the measured signals. For example, the classical increase in heart rate can no longer be attributed solely to stress and could be caused by the activity itself. This makes the development of mobile passive B/BCIs challenging. In this paper, we introduce PASS, a multimodal database of Physical Activity and StresS collected from 48 participants. Participants performed tasks of varying stress levels at three different activity levels and provided quantitative ratings of their perceived stress and fatigue levels. To manipulate stress, two video games (i.e., a calm exploration game and a survival game) were used. Peripheral physical activity (electrocardiography, electrodermal activity, breathing, skin temperature) as well as cerebral activity (electroencephalography) were measured throughout the experiment. A complete description of the experimental protocol is provided and preliminary analyses are performed to investigate the physiological reactions to stress in the presence of physical activity. The PASS database, including raw data and subjective ratings has been made available to the research community at http://musaelab.ca/pass-database/. It is hoped that this database will help advance mobile passive B/BCIs for use in everyday settings.

## 1. Introduction

Brain-computer interfaces (BCIs) are systems that provide communication and control abilities to users without relying on the brain's normal output pathways (Wolpaw et al., 2000). BCIs are typically divided into two categories (Tan and Nijholt, 2010): active or passive. Active BCIs are systems where users must actively modulate their brain responses in order to control the BCI. Passive BCIs, in turn, monitor the user's implicit states, thus do not require the user to perform any specific task. More recently, some researchers have started to use the term “Body/brain-computer interfaces” (B/BCIs) to extend the inputs of BCI to the rest of the physiological system (e.g., Feng et al., 2016).

Physiological measures and passive body/brain-computer interfaces offer tremendous possibilities for monitoring individual functional states. In recent years, several works have shown that physiological measures can be used to assess e.g., the operator functional state of workers (i.e., workload, stress, fatigue), videogame player fun level, or even health markers (Banaee et al., 2013; Gagnon et al., 2016; Harrivel et al., 2017; Fortin-Côté et al., 2018). Moreover, it has been demonstrated that such assessment can be leveraged to augment interactions with intelligent systems, such as adaptive videogames or adaptive workload management systems (Parnandi and Gutierrez-Osuna, 2015; Aricò et al., 2016). Wearables further push this progress by increasing portability and accessibility of neurophysiological measures, while reducing the cost associated with such systems.

There are many challenges, however, with relying on neurophysiological measures and passive B/BCIs in realistic settings where the user is mobile and multi-tasking. The first relates to the question of multidimensionality of psychological states (Matthews et al., 2015) where different emotions and psychological conditions are combined. While the multidimensionality of psychological states can be well-captured with questionnaires, it becomes harder with metrics derived from neurophysiological models. One example of this is the overlapping of e.g., physical activity and stress on heart rate and heart rate variability. An additional challenge lies on the artifacts that are generated once experiments are performed outside controlled laboratory settings with sensors that are sensitive to e.g., movement artifacts (Sun et al., 2010; Falk et al., 2016).

The first goal of this project is to provide a multimodal dataset where affective stress and physical activity are both modulated. To date, there are no publicly-available datasets that explore the concurrent modulation of affective stress and physical activity and the impact it has on physiological measures and on artifact generation. We aim to fill this gap. The second goal of this article is to provide a dataset that mimics realistic settings to support “in-the-wild” B/BCI development. To do so, we used a realistic task setting (i.e., playing video games) and used off-the-shelf wearable devices. Modalities used in this study include electroencephalography, cardiac activity, electrodermal activity, breathing information, and skin temperature.

In this paper, we describe PASS, a multimodal database of Physical Activity and StresS. Here, we present the experimental protocol used, descriptive statistics of the recorded neurophysiological signals under the varying conditions, and also introduce preliminary results on the use of machine learning to model stress that is robust to different physical activity confounding factors. The database has been made publicly available at http://musaelab.ca/pass-database/, along with stress and physical fatigue questionnaire responses provided by the participants.

In the remainder of this paper, we first provide background on the theory and physiological measures of stress in section 2, followed by a description of the current challenges in stress monitoring in section 3. Next, a full description of the experimental design and the methodology used to perform the data collection is presented in section 4. Validation of the dataset is presented in section 5, including analyses on the physiological and subjective data gathered. Results are then discussed in section 6 and conclusions drawn in section 7.

## 2. Background

### 2.1. Theory of Stress

Stress is a psychological concept that has received a tremendous level of scientific attention throughout its history. One could argue that this attention is well-placed, as stress is well-known to have several negative effects on individual health and performance. While many definitions of stress exist, it can be generally defined as an ensemble of coping responses to react to a perceived threat (Lazarus and Folkman, 1984).

While some amount of stress is inevitable, extended or acute exposure to stress is known to be associated with several health problems such as cardiovascular diseases, respiratory diseases, and autoimmune diseases (Schneiderman et al., 2005). Investigations of occupational stress in many countries have shown that a large proportion of the population is exposed to detrimental levels of stress through their work environment (Jones et al., 2016), increasing absenteeism and turnover intention (Jamal, 2007). Finally, stress is associated with psychological disorders like depression (Caspi et al., 2003).

Besides health considerations, several researchers have described intricate links between stress and human performance. Stress has been shown to influence cognitive performance, such as memory. Authors suggest that high arousal could enhance memory consolidation, but could hamper memory recall (Wolf, 2009). Anxiety is also linked with poorer manual dexterity (Kneller et al., 2012; Skirbekk et al., 2012). In job settings, stress is associated with lower job performance (Jamal, 2007). Despite these results, some findings suggest that stress might be beneficial in some circumstances. Using a crisis management simulation, authors investigated the link between stress (i.e., time pressure) and team communication. They found that stress increases communication quantity and efficiency. They do, however, underline that frequent requests for information are associated with poorer task performance (Pfaff, 2012). Stress also influences academic performance. A recently published longitudinal study showed that children and adolescents undergoing an anxiety treatment therapy were associated with better academic performance (Swan et al., 2018).

In the literature, stress is conceptualized in various ways. First, stress can refer to shorter-term activation, caused by more immediate situations (e.g., solving a problem). On the other hand, stress can also relate to longer-term straining states (i.e., chronic stress, occupational stress), caused by adverse life or job situations (e.g., disease, mourning, layoff) or by prolonged exposure to short-term stress (Schneiderman et al., 2005; Schubert et al., 2009). In experimental settings, most researchers use *validated stressors* to induce stress in participants. For example, the cold-pressor test which requires participants to submerge limbs in near-frozen water for a short period of time has been used in several studies investigating stress (McRae et al., 2006; Duncko et al., 2009; Dierolf et al., 2017).

Validated stressors can elicit two forms of stress: mental stress and affective stress. Mental stress refers to situations that require reflection and problem-solving abilities (Sun et al., 2010; Al-Shargie et al., 2016). For example, the stroop task or mental arithmetic task, are designed to stress individuals by requiring mental effort (Visnovcova et al., 2014). Mental stress is closely tied to the concept of mental workload. Mental workload can be difficult to define (Young et al., 2015). In general, it can be considered as the level of mental resources required to meet a specific performance (Young et al., 2015). On the other hand, affective stress relates to anxiety, fear or discomfort. Such stressors include the Trier Social Stress Task (Kudielka et al., 2007). The Trier Social Stress Task requires individuals to perform tasks, such as oral presentations or mental arithmetic, in front of fake experts. Affective stress is generally associated with emotions of negative valence (Hwang et al., 2018), with various levels of arousal. Therefore, affective stressors also include viewing emotionally loaded stimuli, such as pictures, movies, or sentences (Wolf, 2009).

### 2.2. Physiological Measures of Stress

Stress can be assessed using subjective measures. Various questionnaires have been developed to measure stress related to tasks (Matthews and Campbell, 2010) or anxiety (Spielberger, 2010). Subjective measures have the advantage of being simple and to offer direct access to cognition; however, they are also known to be biased. Furthermore, they require interruptions. Physiological measures, on the other hand, are objective and can be taken continuously, without interruptions. As such, several recent studies have proposed physiology-based models, sometimes achieving fairly high detection accuracy (Smets et al., 2019).

#### 2.2.1. Neurophysiological Measures

Stress generates a wide range of physiological reactions that can be leveraged to measure its intensity in individuals. It can be assessed using electroencephalography (EEG), but elicited patterns are very dependant on the type of stressor used. Task demand and temporal pressure are often associated with a decrease in the alpha band power in various cerebral regions, including frontal, central and parietal, and associated with an increase in theta at frontal and parietal regions (Borghini et al., 2014; Al-Shargie et al., 2016). Individuals performing the Montreal Imaging Stress Task (mental arithmetic combined with negative social feedback) have been shown to exhibit greater relative gamma band power in prefrontal, temporal, and parietal regions (Minguillon et al., 2016). Similarly, the gamma band is associated with worry. Individuals suffering from generalized anxiety disorder undergoing a worry task (self-selected worrying thought) exhibited greater gamma power in temporal and parietal lobes (Oathes et al., 2008). In another study, prefrontal asymmetry of participants performing a virtual reality surveillance task was investigated (Brouwer et al., 2011). During stressful moments (i.e., a bomb explosion combined with negative feedback), alpha asymmetry of prefrontal regions (F7-F8) was significantly higher than during non-stressful moments. Prefrontal asymmetry was also associated with stress in other studies, such as participant performing the Maastricht Acute Stress Task (Quaedflieg et al., 2015). While not investigated directly in studies involving stress, amplitude modulation features of EEG have shown discriminative power for valence and arousal measurement (Clerico et al., 2018), as well as workload (Albuquerque et al., 2018). Stress is also known to influence event-related potentials, for example, during sustained attention tasks (Righi et al., 2009). Apart from EEG, stress can be measured using other neurophysiological measures, such as functional near-infrared spectroscopy (Al-Shargie et al., 2016; Parent et al., 2019a).

#### 2.2.2. Cardiac Measures

Stress is well-known to increase heart rate. Heart rate is often derived from the electrocardiography (ECG) signal. ECG consists of placing electrodes on the skin to measure the voltage difference caused by the electrical activity of the heart. Heart rate can also be measured using photoplethysmography by measuring variations of the light absorption of the skin. Apart from heart rate, stress is known to influence heat-rate variability (Kreibig et al., 2007; Castaldo et al., 2015). Heart rate variability is the analysis of the changes in heart rhythm. Heart rate variability does not usually refer to a specific feature, but a family of features, each describing various aspects of cardiac activity. As such, stress is known to increase the standard deviation of inter-beat intervals (SDNN) or reduce the root mean square of inter-beat intervals (RMSSD) (Castaldo et al., 2015). Stress also influences frequency-domain features of heart rate variability, as the ratio between low and high frequency power (Castaldo et al., 2015). Blood pressure is also influenced by stress. Fear is known to increase both systolic and diastolic blood pressure (Kreibig et al., 2007). In a simulation of computer work containing stressful and non-stressful sessions, it was shown that blood pressure increased during work sessions compared to rest, but did not decrease during non-stressful sessions (Hjortskov et al., 2004).

#### 2.2.3. Breathing Measures

Breathing rate increases under stress (Rainville et al., 2006; Homma and Masaoka, 2008). Furthermore, anxious individuals tend to breathe faster during anticipatory stress than less anxious individuals (Homma and Masaoka, 2008). Studies have also shown that respiratory variability is higher and more random during mental stress and worry (Vlemincx et al., 2013). In the same line of thought, fear is associated with higher standard deviation of breathing amplitude (Rainville et al., 2006). Sighing seems more present during stress (Vlemincx et al., 2013). It is suggested that sighing might act as a reset to irregular respiration pattern encountered during stress.

#### 2.2.4. Electrodermal Measures

Stress also has effects on sweating, which can be measured using electrodermal activity (EDA). EDA is described as the electrical conductance of the skin, which is modulated by the level of sweat. Sweat is well-known to be influenced by physical activity. However, it is suggested that sweat glands are controlled by the sympathetic system. EDA is thus considered as a proxy to observe the sympathetic activation of individuals. Besides the electrodermal level (i.e., the “amount” of sweat on the skin), EDA can be described in greater details by analyzing electrodermal responses. Electrodermal responses are brief “peaks” of sweat that occurs in response to a stimulus. They can be specific (i.e., related to a known event) or non-specific (Boucsein, 2012). Typically, short-term stressors used in laboratory settings, such as the cold pressor or stroop task, or fearful states tend to increase electrodermal level, non-specific response frequency, as well as response amplitude (Kreibig et al., 2007; Reinhardt et al., 2012; Posada-Quintero et al., 2016b, 2018a). While still not very common, some authors have investigated frequency domain features of the EDA. Overall, results suggest that the stressors influence mostly the 0.045 to 0.15 Hz band (Posada-Quintero et al., 2016a). Frequency domain features of EDA are said to be sometimes more sensitive to stress than classical time-domain features (Posada-Quintero et al., 2016b, 2018a).

#### 2.2.5. Thermal Measures

In reaction to stress, mammals, including humans, typically have a reduced temperature in peripheral regions, while the temperature of the face and core region rises (Marazziti et al., 1992; Vianna and Carrive, 2005; Kreibig et al., 2007; Nakamura, 2011). It is theorized that this reaction is caused by a constriction of the peripheral arterioles, which could reduce blood loss if a wound occurred.

## 3. Current Challenges

### 3.1. Multidimensionality of Stress

It is challenging to fully separate mental stress from affective stress, as all mental tasks will still trigger even a low amount of anxiety in individuals. Conversely, affective stress will probably trigger even a small amount of mental activity, whether it is due to assessing the threat, planning a response or simply diverting attention to less stressful states. Yet, both types of stress have different implications. For example, authors suggest that mental forms of stress (like engagement) correlate with working memory performance while affective forms of stress (like distress) negatively correlate with performance (Qin et al., 2009; Matthews and Campbell, 2010). On the physiological level, it is suggested that mental effort is associated with sympatho-adrenal-medullary axis (epinephrine and norepinephrine) while affective stressors are more associated with the hypothalamus-pituitary-adrenal (cortisol) axis. While there is not an extensive amount of literature to support this, it can be surmised that high mental stress with minimal affective stress might lead to positive outcomes (like task completion) while high affective stress without much mental activation is not beneficial in any way. This view was supported by some authors investigating physiological differences between mental effort and distress (Frankenhaeuser, 1986; Gaillard and Wientjes, 1994; Matthews et al., 2015) and does, to a certain extent, resemble the eustress/distress dissociation proposed by Hans Seyle in his classical work on stress (Selye, 1985).

Subjective tools attempt to distinguish between these nuances of stress. The NASA-TLX questionnaire, for example, features a “Frustration” axis, covering affective load among more cognitive ones (Hart, 2006). The Dundee Stress State Questionnaire also distinguishes more mental stress (i.e., engagement) from affective forms (i.e., distress, worry) (Matthews and Campbell, 2010). In contrast, physiological measures of stress, despite being well-documented, are rarely interpreted in a multidimensional way (Matthews et al., 2015). Distinguishing mental and affective stress using physiology remains a challenge today. The separation of mental and affective stress goes beyond the scope of this database description work, thus henceforth, the term “stress” will be used to comprise their combined effects. Notwithstanding, future work can explore such separation with multimodal tools (e.g., Parent et al., 2019b).

### 3.2. Stress Detection in Laboratory and Ambulatory Settings

Given the numerous effects of stress on the human body, research has focused on trying to propose models to detect stress based on physiology. In a recent article (Smets et al., 2019), the authors reviewed 25 papers that investigated this research question over the last several years. Comparing the performance of each model investigated in these studies can be difficult as several factors can differ between studies. First, as detailed previously, different stressors can be used. Second, models use different physiological modalities and, in some cases, different combinations of modalities. Models also differ in terms of classification scheme (i.e., within participants, between participants) and classification levels. Most studies propose models that distinguish between a resting state and a stressful task. However, some studies attempt to classify multiple levels of stress (e.g., low, medium, high) and others use regression models to measure a stress level (e.g., Hovsepian et al., 2015). Finally, as described by Smets et al. (2019), the majority of papers focus on laboratory settings, while only a select few have attempted to detect stress in ambulatory settings.

In laboratory settings, classification accuracy of stress detection models can reach fairly high levels. In a recent example, researchers used a portable wristband, recording heart and electrodermal activity, to detect affective stress induced by the Trier Social Stress Task. They reported achieving an area under the receiver operating characteristic curve of 0.87 (Ollander et al., 2016). In another case, researchers used ECG and EEG to classify the affective state of individuals playing a survival horror game (Vachiratamporn et al., 2013). Six different affective states were classified. Authors reported up to 90% classification accuracy using ECG and up to 73% using EEG. In a recent study, the Muse headband (i.e., the same low-cost EEG system used in this study) was used to classify the subjective stress level of participants (Arsalan et al., 2019). Authors reported accuracy as high as 92% on a two-class classification task. Finally, by using EEG and near-infrared spectroscopy, detection of mental stressors with accuracy near 95% (i.e., distinguishing between control and stress) has been reported in Al-Shargie et al. (2016).

In ambulatory settings, however, performance is usually lower. Nonetheless, the topic gained scientific attention in the last few years, improving the potential of ambulant stress detection models. In two recent examples, portables sensors (i.e., a chest strap, a wristband) were used to detect stress in ambulatory settings (Hovsepian et al., 2015; Gjoreski et al., 2016). Models reached, respectively, a 0.72 correlation coefficient or 76% classification accuracy at detecting self-reported stress (i.e., two-class classification). In a recent study, EEG asymmetry was used to monitor arousal and valence of individuals in the presence of physical activity (i.e., construction workers) (Hwang et al., 2018). While comparison to ground truth is difficult in naturalistic situations, the authors suggest that this method had potential to assess emotional state of individuals, especially for valence detection.

Despite advances in ambulant stress detection, several challenges are still in the way of a highly robust stress detection model. As suggested by Smets et al. (2019), movement and physical activity are the most obvious limitations of stress detection models. Some models are configured to not predict stress if physical activity is detected (Hovsepian et al., 2015), thus not only biasing error rate measures, but also preventing stress detection in the presence of physical activity. Other models are configured to receive contextual data (such as physical activity), improving accuracy in exchange of manual input in the model (Gjoreski et al., 2016). However, very few papers have investigated stress detection in the presence of varying levels of physical activity.

Movement and physical activity affect physiological measures in three different ways. First, physiological measures are influenced by the direct consequences of physical activity. When individuals start to perform physical activity, the body triggers a series of physiological mechanisms to shift from a rest state to an active state. The most obvious example is the increase in heart rate caused by physical exertion (Bernardi et al., 1996). Since physical activity requires energy, the heart must beat faster to deliver more supplies to muscle cells, fetch more oxygen and reject more CO_2_ in the lungs. The skin sweat will also be increased to dissipate excess of heat caused by physical activity (Neto et al., 2010). The response of the central system will also be affected, as some areas of the brain will be required to coordinate limb movements.

Second, physiological measures are influenced by shifts in psychological states that come with physical activity. For example, it has been shown that performing physical tasks, such as lifting boxes, will draw mental resources (DiDomenico and Nussbaum, 2008). In relation with this paper, there is also some evidence that physical activity can reduce long term stress and anxiety (Pedersen and Saltin, 2015). While scientific attention is mostly oriented towards long-term benefits of physical exercise regarding stress, evidence also suggests the presence of short-term effects (Salmon, 2001). Individuals are most likely to report having a better mood immediately after exercise. Some factors modulate this relationship. Having a poor mood before exercise usually causes a sharper improvement in mood after exercise. On the other hand, performing at higher intensity than habitual level can deteriorate mood.

Finally, movement and physical activity alter physiological recordings through noise or signal loss. If the device uses electrodes (e.g., EEG, ECG, EDA), these might lose contact with the skin, briefly or continuously, and alter the measured signal (Castellanos and Makarov, 2006; Gwin et al., 2010). The nature of the physical task might also displace, disable or even damage sensors. If the data is transmitted wirelessly, signal loss might be encountered when the distance between the emitter and the receiver is too high or when an obstacle is present between them.

## 4. Methods and Materials

### 4.1. Motivation and Overview

The experiment discussed in this paper sought to elicit affective stress. Common stressors used in psychophysiology (e.g., Stroop task, n-back task) were excluded since they were not sufficiently independent from mental stress. In a similar way, time pressure (sometime used as a stressor) was also discarded since higher time pressure can sometimes lead to higher mental effort. To support “in-the-wild” B/BCI development, we also sought to use a realistic task setting. Therefore, a survival video game was selected as a stressor. Video games have already been used in affect research, and, as in our case, in combination with physiological measures. Survival video games also allow for a short-duration experimental design (compared to studies that focus on more chronic, long-term stressors).

More specifically, the experiment consisted of playing video games while pedaling on a stationary bike. Two experimental variables were manipulated: stress and physical activity intensity. There were two stress levels (no stress/stressful) and three physical activity levels (0, 18, 24 km/h). Participants performed all six combinations in counterbalanced order. Each trial lasted 10 min. Physiological activity and subjective ratings were recorded throughout the experiment. The following sections will provide more details about the experimental design.

### 4.2. Stress Manipulation

**Figure 1:**
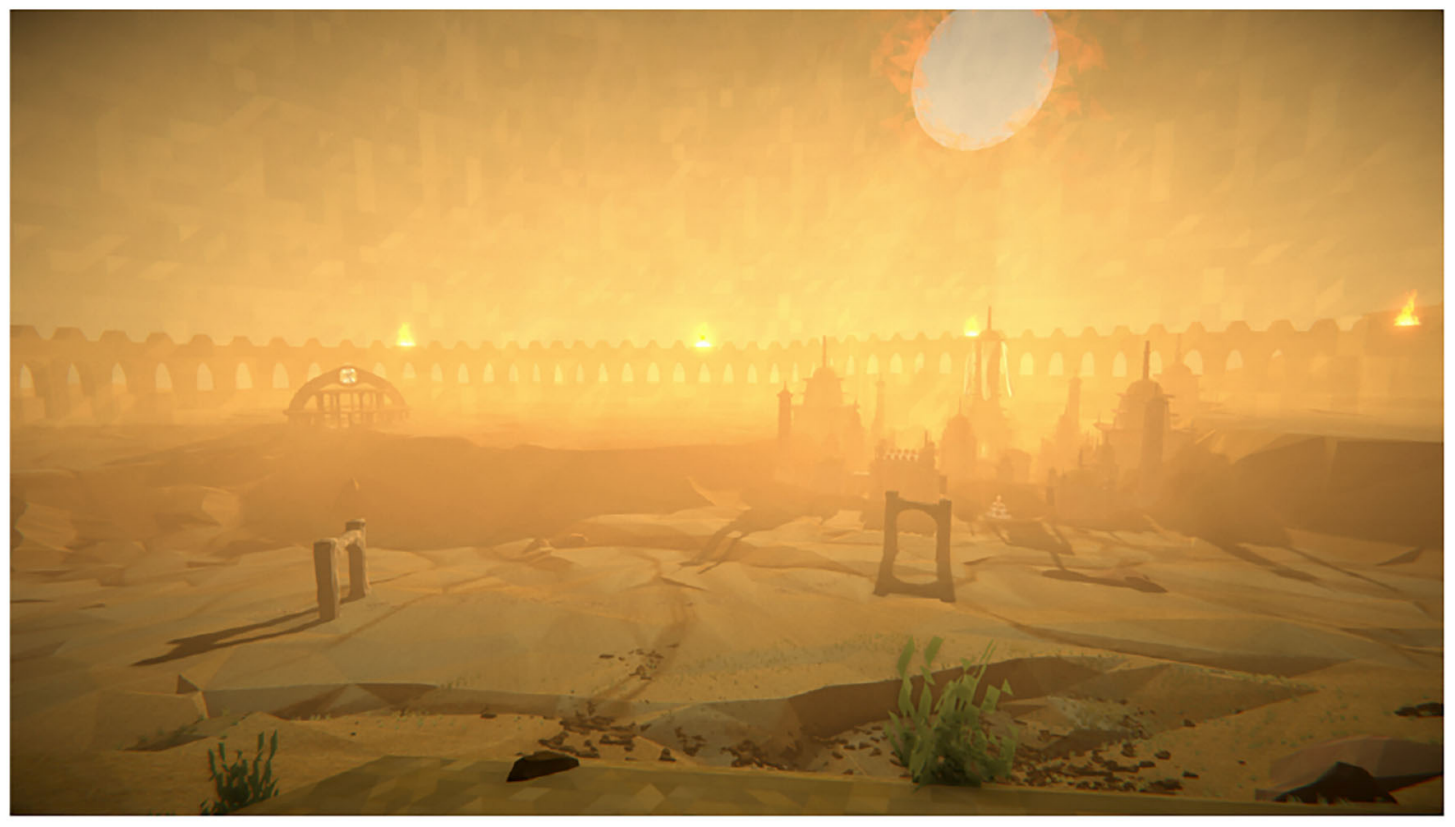
Screenshot from TIMEframe.

Stress was modulated by switching between two video games: a non-stressful one, serving as a control condition, and a stressful one. The non-stressful game used was TIMEframe. TIMEframe is a commercially available exploration/puzzle game developed by Random Seed Games (Random Seed Games, 2015). In TIMEframe, players must explore ruins of an abandoned city and find artifacts. The game is played from a first-person perspective and controls are similar to other first-person games. Several elements made TIMEframe a prime choice for a non-stressful game. First, there are no significant threats in the game, as the players' personas can not be harmed or die. Also, the music is soft and the environment is bright and peaceful. To further decrease stress, players were told that the number of artifacts they found would not matter and would not be recorded. The game was controlled with an Xbox One controller. Figure 1 shows a screenshot of the game.

**Figure 2:**
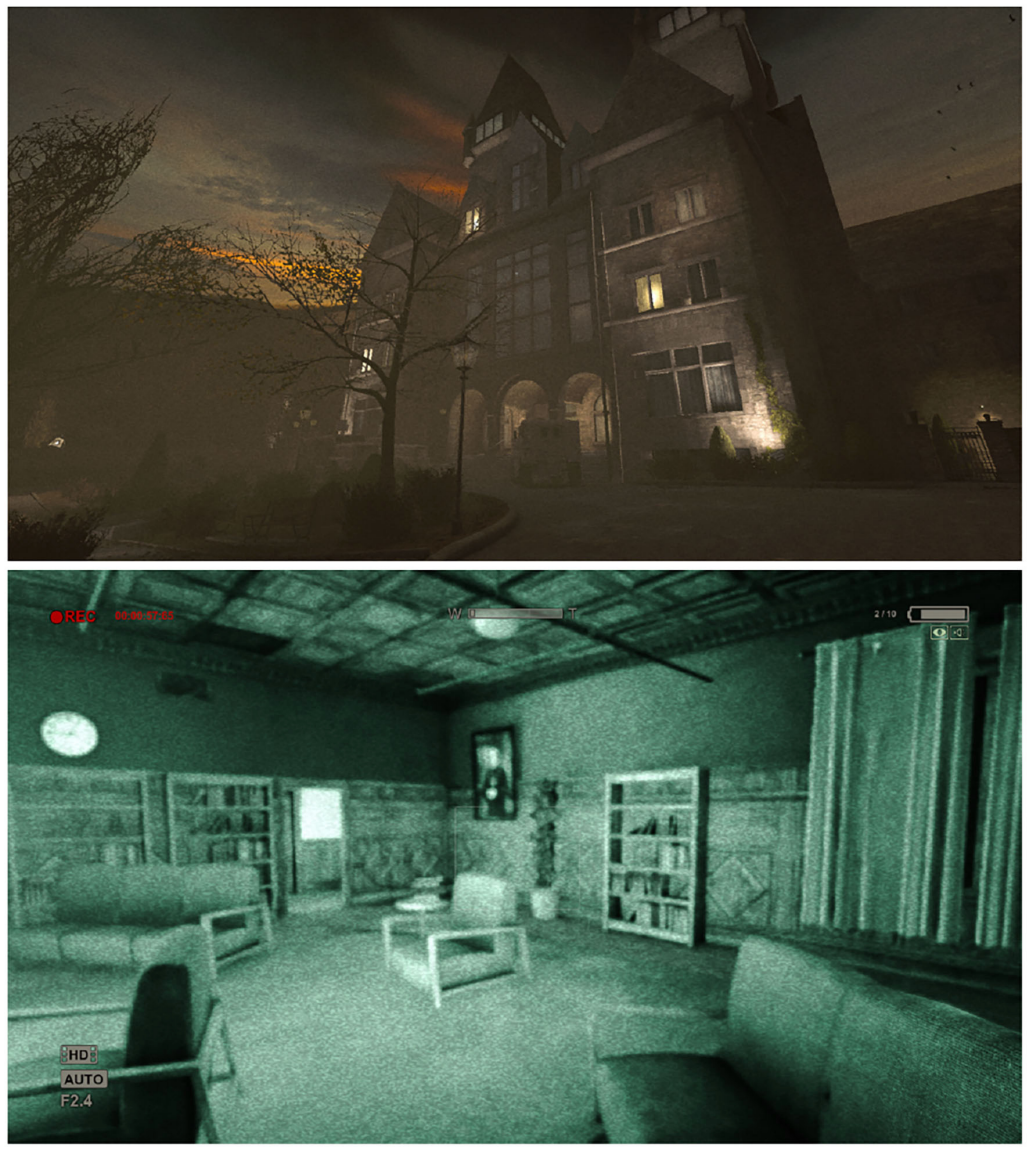
Screenshot from Outlast showing normal **(top)** and night vision modes **(bottom)**.

On the other hand, the stressful game used was Outlast. Outlast is a commercially available survival game developed by Red Barrels (Red Barrels Games, 2013). Like TIMEframe, Outlast is viewed from a first-person perspective and controlled in a similar fashion (albeit, slightly more complex than TIMEframe). The goal of the game is to navigate in a creepy asylum and evade capture/harm by its dangerous inmates. In Outlast, players cannot fight, they can only avoid, escape or hide from enemies. The game features several elements to increase stress, such as an eerie music/sound design and a horror-style environment. Some in-game areas are also poorly lit, requiring players to use a limited night vision mode. The experiment room ambient light was also dimmed to further increase stress. Outlast is deterministic and features a fairly linear playthrough, increasing the similitude of experience between participants. Once again, the game was played with an Xbox One controller. Figure 2 depicts two screenshots of the game, one showing normal and the other (bottom) night vision mode.

Video games have been used in psychophysiological studies for some years (e.g., Carroll et al., 1987). Still, their potential as stressors is fairly unexplored. One study found that video games can elicit similar effects to common stressors such as the Trier Social Stress Task (Guitard et al., 2010). On the other hand, some studies have suggested that they have the potential to stress individuals in a way that differ from commonly used stressors, calling for more investigation on the subject (Porter and Goolkasian, 2019). The TIMEframe/Outlast manipulation was designed with stress in mind. However, this manipulation might have elicited other aspects of cognition, such as workload, engagement or enjoyment.

### 4.3. Physical Activity Manipulation

**Figure 3:**
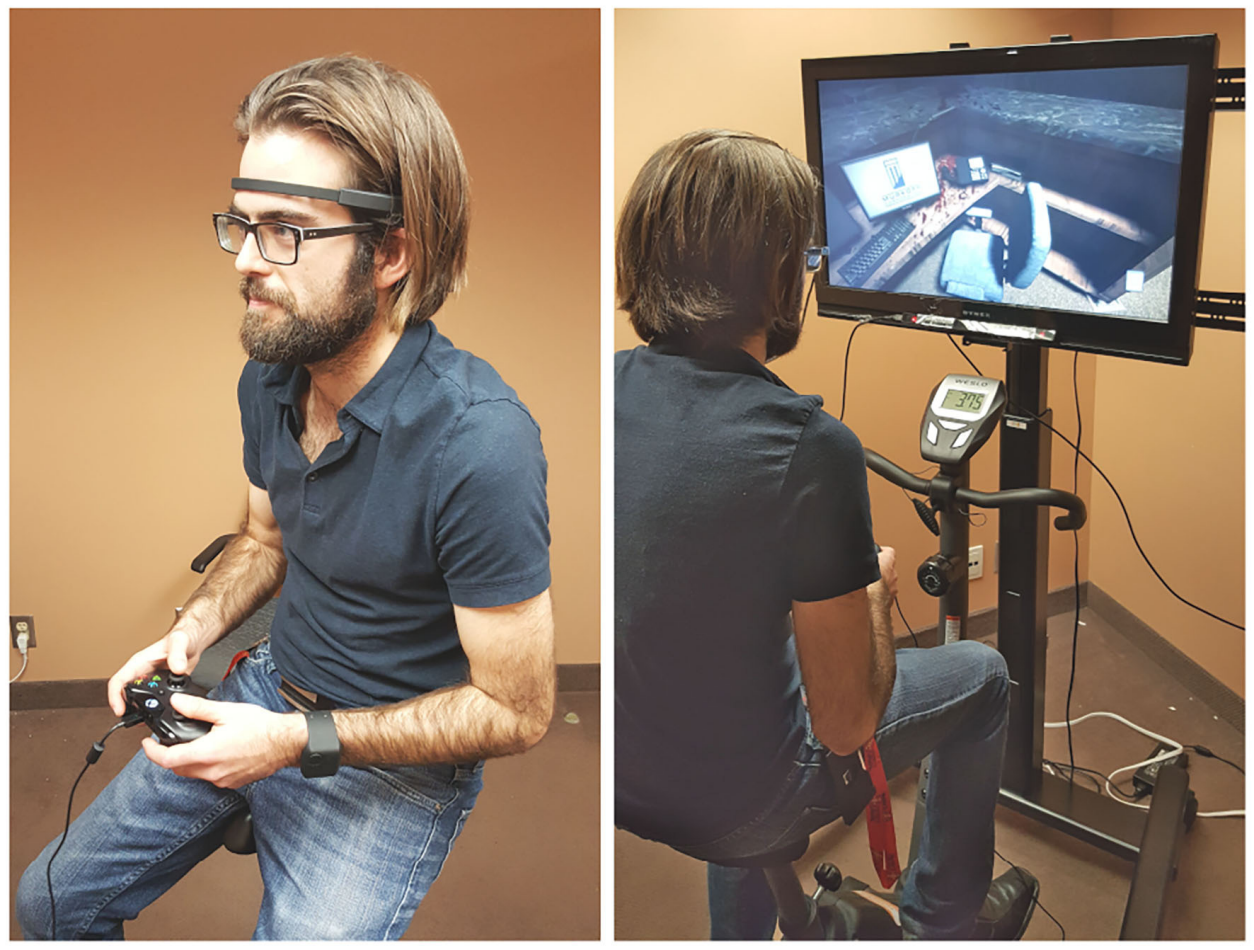
Experimental setup from the front **(left)**. Experimental setup from the back **(right)**. BioHarness 3 not shown since worn under the shirt.

Physical activity was induced by asking participants to pedal on a stationary bike. The bike used featured an adjustable seat, a resistance setting and a display. The resistance was set to its minimum value (no resistance) to maximize reproducibility of the experiment. Since the participant held a controller throughout the experiment, the bike handles were unused and were flipped (see Figure 3). The bike display was set to show speed (in km/h).

Physical activity was modulated by changing the required speed at which participants pedaled on the bike. Our ultimate goal was to induce physical activity and artifacts to the sensors, though not to a point of making the data completely unusable. Therefore, three levels of physical activity were used. In the first level, the speed was 0 km/h; participants were simply told to sit on the bike and not pedal. At the second level, participants were told to maintain a target speed of 18 km/h. At the third level, the target speed was raised to 24 km/h. During a pilot study, we found that these speeds provided the optimal trade-off in signal quality and movement artifact generation. While most of the physical effort was made by the legs of participants, the fact that they had to hold the controller in their hand inevitably created head sways and movements. Since it can be difficult to maintain a constant speed, a tolerance of ±2 km/h was allowed. Experimenters warned participants who drifted from the target speed during the trials. Despite focusing on the video games, the pilot study showed that most participants were able to maintain speed within the tolerance levels.

### 4.4. Counterbalancing

Each participant completed all six combinations of stress (no stress, stressful) and physical activity (0, 18, 24 km/h). The order of these conditions was counterbalanced and pseudo-randomized. All conditions from the same video game were performed subsequently. This was designed to avoid constant psychophysiological shifts between calm and stressful states. Doing so also allowed participants to learn the controls of one video game at a time instead of two. Each condition lasted 10 min. In TIMEframe, there were no differences in the three times participants played the game except that participants were told not to seek the same artifacts as previous sessions. For Outlast, a different scene (start point) was selected for each of the three times that participants played the game. Table 1 describes the three in-game start points. While it is technically possible for a participant to reach another condition start point before finishing the conditions, they were sufficiently distanced, so it never happened for any participant.

**Table 1:** Description of the three starting points used in Outlast.

**Scene**	**In-game description**
Admin	After being thrown out the window (admin block).
Ward	After waking up in the male ward cell.
Sewers	After the valve puzzle (chased by Chris).

### 4.5. Physiological Measures

As mentioned earlier, one of the goals of this study is to provide a database that is captured using off-the-shelf devices. Four wearable physiological devices were used in this study. A BioHarness 3 was used to measure cardiac and respiratory activity. The BioHarness 3 is a chest strap worn directly on the skin. It measures heart activity through ECG at a sampling frequency of 250 Hz. Respiration is recorded by measuring the extension of the chest strap (18 Hz). Besides ECG and respiration, 3-axis acceleration (100 Hz) is also recorded by the device (these signals were not used in the current study). An E4 wristband was also used. The E4 records blood volume pulse through photoplethysmography (64 Hz), as well as skin temperature (4 Hz). Two electrodes, located inside the bracelet, also record galvanic skin responses (4 Hz). Cerebral activity was recorded using a Muse headband. This headband records EEG activity using 4 electrodes (TP9, AF7, AF8, and TP10) with reference to Fpz, at a 220 Hz sampling rate. From our past experience with the Muse headband, we have found that re-referencing the signals to electrodes over the temporal lobes (TP9 or TP10) could negatively impact the EEG recordings, as these signals are more prone to movement artifacts. Therefore, the acquired EEG signals were not re-referenced prior to analyses. The BioHarness 3, E4 and Muse data were streamed to a nearby laptop using Bluetooth protocol. Data was recorded using the MuSAE Lab EEG Server (MuLES), which was also used to send triggers marking the beginning and end of trials (Cassani et al., 2015).

### 4.6. Subjective Measures

Beside physiological measures, subjective measures were also collected. Two questionnaires were used: the NASA-TLX and the BORG. NASA-TLX is a questionnaire designed to measure workload of individuals. The original version features six questions, which must be answered on a 21-point Likert scale. In this experiment, two additional questions related to stress and fear were added to suit the research questions of the project. Table 2 shows the extra questions used. These questions were asked in French to all participants who spoke French as their first language.

**Table 2:** Stress and fear questions added to the NASA-TLX questionnaire.

**Label (English)**	**Question (English)**
Stress	How stressful was the task?
Fear	How scary was the task?
**Label (French)**	**Question (French)**
Stress	À quel point la tâche était-elle stressante?
Peur	À quel point la tâche était-elle effrayante?

### 4.7. Experimental Procedure

Forty-eight participants were invited to perform an experiment at Université Laval (Quebec City, Canada). Participants were recruited using mailing lists. Candidates with heart or respiratory problems or having neurological/psychological disorders were excluded from the experiment. Given the nature of the stressor, precautions were taken to make sure participants were comfortable playing Outlast. People with a history of aversive reaction to horror (e.g., panic attacks, related phobia or just unease with featured themes) were excluded from the experiment. To avoid bias, participants who played either TIMEframe or Outlast in the past could not participate in the study. During the tutorial, participants were given warning about the expected features of the stressor. The tutorial reminded participants that they could interrupt their involvement at any moment without prejudice. Experimenters were also trained to check participant's well-being during Outlast's practice, game sessions and breaks. The experimental protocol was approved by the Ethics Review Boards of the Institut national de la recherche scientifique (INRS; Reference number: CER-16-425), the PERFORM Center (Concordia University; Reference number: 30006772) and Université Laval (Reference number: 2016-274). Participants gave written consent to participate in the study and were remunerated for their time.

Participants were greeted and invited to fill a consent form and demographic questionnaires. After these, they were briefed on the experimental procedure. Once done, physiological sensors were donned and configured in a particular order. The BioHarness 3 chest-strap (Zephyr, USA) and the E4 wristband (Empatica, USA) were donned first on the participant, as they were deemed less susceptible to be disrupted during the installation of the other devices. Afterward, participants were invited to adjust the height of the stationary bicycle seat. The TV monitor height was then adjusted in order for the screen to be at the participant's eye level. Finally, the Muse headband (Interaxon, Canada) was donned on participants forehead. The experimenter made sure the headband was positioned correctly and was comfortable for the participant.

Participants were then invited to perform a task tutorial (in the form of a PowerPoint presentation). In order to avoid information overload, this tutorial only contained the information about the first video game they were set to play. After the tutorial, participants were invited to practice the first game they were set to play in order to become familiar with the controls. This lasted between 5 and 15 min, depending on participants. Once done, participants completed their first three conditions (the three physical activity levels for the first game). Each of these conditions lasted 10 min. A 2-min baseline was performed before each condition. This baseline consisted in performing the same level of physical activity as the upcoming condition, but without playing any game. Conditions were performed with minimal disruption. The experimenter warned participants who pedaled too slowly or too fast. Additionally, the experimenter tipped players who got stuck for too long in a specific spot. After each condition, participants were invited to complete the two subjective measures questionnaires (NASA-TLX and BORG) and take a short break (roughly 5 min).

**Figure 4:**
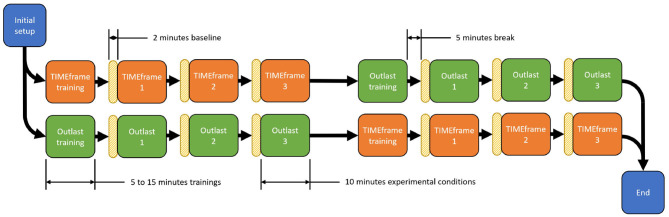
Diagram showing the experimental sequence. After the initial setup, participants completed a training of their first game (5–15 min). They then performed the 3 levels of physical activity for this game. The order of physical activity level was counterbalanced. Each condition lasted 10 min. A 5-min break was inserted between conditions. Before every condition, participants did a 2-min baseline in which they did the same level of physical activity as the upcoming condition, but without playing the game. Half of the participants began with TIMEframe. The other half began with Outlast.

Once the three conditions of the first game were completed, participants were presented the tutorial of the second game and performed the remaining three conditions. Two reasons motivated a design in which all conditions of the same game were done subsequently like this. First, we wanted to avoid overloading or confusing participants with shifting game mechanics and controls. Second, we wanted to minimize the lagged effects of stress. Stress is known to influence physiological response even after the stressor is removed (Tassorelli et al., 1995; Qin et al., 2009). While these lagged effects cannot be fully removed from the design, the 5 min breaks between conditions and the non-alternating game conditions helped alleviate this. Figure 3 shows the experimental setup used. Figure 4 shows the experimental sequence.

### 4.8. Physiological Signal Recording

Physiological signals were recorded using the MuSAE Lab EEG Server (MuLES) software (Cassani et al., 2015). MuLES is a LabVIEW software designed to ease simultaneous recording of EEG and other physiological signals. It allows data acquisition of various devices as well as real-time streaming of physiological signal. In our case, data was streamed to a custom-made MATLAB script designed to input markers delimiting the beginning and the end of all experiment trials. Once the data collection finished, a lab assistant manually verified all markers to make sure they correctly matched the experimental trials.

### 4.9. Signal Processing and Feature Calculation

Physiological signals were loaded in MATLAB using a custom-made parser and trimmed to keep only the relevant parts (baseline and trials). For trials, signals were trimmed into two epochs of 5 min. Baselines were trimmed into epochs of variable length (more or less 2 min).

For the processing of EEG signals, previous works (e.g., Snyder et al., 2015; Bono et al., 2016) have shown that artifact removal methods based on the independent component analysis (ICA) can be successfully employed to enhance EEG data in scenarios where artifacts due to physical activity are present. Among these methods, the wavelet-enhanced ICA method, (Castellanos and Makarov, 2006), allows automated artifact removal, and has been proven effective in different scenarios where EEG was acquired with low-density wearable devices (e.g., Cassani et al., 2017; Rosanne et al., 2019). The parameter used for the wICA method in our experiments relied in a threshold *K* = 1 set empirically.

For EEG feature extraction, prefrontal (AF7–AF8) alpha and theta absolute power, and relative gamma power (all locations) were computed since they are known to be associated with stress (Borghini et al., 2014; Minguillon et al., 2016). Prefrontal asymmetry has also been found to be associated with stress (Brouwer et al., 2011) and was computed here between AF8 and AF7. Asymmetry between TP9 and TP10 was also computed for exploratory purposes. Coherence (alpha and beta band) have also been associated with stress in parietal and occipital regions (Giannakakis et al., 2015). As such, it was decided to compute coherence in the closest region available (TP9–TP10) in four frequency subbands (alpha, beta, gamma, and theta). Finally, amplitude modulation features were also computed as per (Falk et al., 2012). Focus is placed here on two specific amplitude modulation features, namely beta modulated by delta (represented as beta-delta) and gamma-delta, given insights reported in Falk et al. (2012), Clerico et al. (2018), and Minguillon et al. (2016). Table 3 summarizes the EEG features computed, as well as our hypotheses of expected behavior under stress. Expected behavior does not account for possible effects of physical activity.

**Table 3:** EEG features and their expected behavior under stress.

**Type**	**Feature**	**Effect**
Absolute power	Alpha (AF7)	↓ (Borghini et al., 2014)
	Theta (AF7)	↑ (Borghini et al., 2014)
	Alpha (AF8)	↓ (Borghini et al., 2014)
	Theta (AF8)	↑ (Borghini et al., 2014)
Relative power	Gamma (AF7)	↑ (Minguillon et al., 2016)
	Gamma (AF8)	↑ (Minguillon et al., 2016)
	Gamma (TP9)	↑ (Minguillon et al., 2016)
	Gamma (TP10)	↑ (Minguillon et al., 2016)
Asymmetry	Alpha (AF7-AF8)	↑ (Brouwer et al., 2011)
	Alpha (TP9-TP10)	–
Coherence	Alpha (TP9-TP10)	↑ (Giannakakis et al., 2015)
	Beta (TP9-TP10)	↓ (Giannakakis et al., 2015)
	Gamma (TP9-TP10)	–
	Theta (TP9-TP10)	–
Amplitude modulation	Beta-delta (AF7)	–
	Gamma-delta (AF7)	–
	Beta-delta (AF8)	–
	Gamma-delta (AF8)	–
	Beta-delta (TP9)	–
	Gamma-delta (TP9)	–
	Beta-delta (TP10)	–
	Gamma-delta (TP10)	–

*Expected behaviors do not account for possible effects of physical activity*.

For the ECG signals, in turn, a variation of the Pan-Tompkins algorithm was used to obtain the interbeat interval time series (Behar et al., 2018). Interbeat intervals were subsequently processed to remove outliers and improbable points. Heart rate variability features, frequently investigated as correlates of stress, were then computed (Castaldo et al., 2015). These features include the heart rate, the standard deviation of interbeat intervals, the power of the high frequency band, and the low-frequency to high-frequency (LF/HF) ratio. Moreover, the breathing signal from the BioHarness 3 was downsampled from 18 to 6 Hz and filtered to remove noise (low-pass, Chebychev, 2 Hz, 8th order). Features previously shown to be modulated by stress were then computed, including breathing rate (computed by counting the peaks of the filtered signals); breathing variability, computed using sample entropy (*m* = 2, *r* = 0.5) (Vlemincx et al., 2013); and sigh rate, where a sigh is defined as a breath where the amplitude exceeded one standard deviation of the normal breathing amplitude for the condition.

From the E4 wristband, the electrodermal signal was first filtered (low-pass, Chebychev, 1 Hz, 8th order) and the features computed include the electrodermal level (normalized average of the baselines) and the number of electrodermal responses (Boucsein, 2012). In addition, relative low frequency power (0.045–0.15 Hz, LF power) was also computed since recent works suggest that it might be associated with stress (Posada-Quintero et al., 2016a). The E4 wristband was also used to measure temperature. No particular processing was performed on the skin temperature signal. Since stress is known to affect temperature, it was decided to compute the average temperature level and the delta (difference between the end and the initial temperature of a condition) temperature (Kreibig et al., 2007). Moreover, blood volume pulse level was normalized in reference to the average of all baselines of each participant. The minimum and maximum blood volume pulse levels were computed to approximate relative diastolic and systolic pressures. Table 4 summarizes the peripheral features computed, as well as our hypotheses of expected behavior under stress. As previously, expected behaviors do not account for possible effects of physical activity.

**Table 4:** Peripheral features and their expected behavior under stress.

**Modality**	**Feature**	**Effect**
Heart	Heart rate	↑ (Castaldo et al., 2015)
	SDNN	↑ (Castaldo et al., 2015)
	HF power	↓ (Castaldo et al., 2015)
	LF/HF	↑ (Castaldo et al., 2015)
Breathing	Breathing rate	↑ (Rainville et al., 2006)
	Variability	↑ (Vlemincx et al., 2013)
	Sigh rate	↑ (Vlemincx et al., 2013)
Electrodermal	Level	↑ (Reinhardt et al., 2012)
	Number of responses	↑ (Reinhardt et al., 2012)
	Rel. LF power	↑ (Posada-Quintero et al., 2016a)
Temperature	Average temperature	↓ (Kreibig et al., 2007)
	Temperature delta	↓ (Kreibig et al., 2007)
Blood volume pulse	Minimum BVP	↑ (Kreibig et al., 2007)
	Maximum BVP	↑ (Kreibig et al., 2007)

*Expected behaviors does not account for possible effects of physical activity*.

### 4.10. Database Availability

The PASS database is part of a larger project on operator functional state monitoring aimed at building models that take into account mental workload, stress and physical fatigue. In a related work, we describe the WAUC dataset, which presents an experimental protocol to modulate mental workload and physical activity (Albuquerque et al., submitted). Both datasets are available online for download at http://musaelab.ca/pass-database/. Both the PASS and WAUC databases include raw physiological signals, subjective responses, and additional documentation, such as markers information.

### 4.11. Modeling

To assess the discriminatory power of the explored features, machine learning models were developed for stress level classification, i.e., classifying between no-stress (TIMEframe) and stress (Outlast) conditions. All physical activity levels were combined in our analyses. This was done in order to see if it was possible to classify stress even if the current level of physical activity is unknown by the classifier. Accounting for missing data, there were 264 samples for TIMEframe and 248 samples for Outlast. Here, a support vector classifier was used (Smets et al., 2019) and two testing schemes were implemented: k-fold and leave-one-participant-out (LOPO).

Both the k-fold and the LOPO scheme used a nested cross-validation scheme. For the k-fold, samples were folded in five-folds for testing. The remaining 4 folds of the samples (for each testing fold) were subdivided again into five-folds to perform the validation. One fifth of these were used for validation. The rest was used for training. For the LOPO scheme, samples were folded per participant for testing. One fifth of the remaining participants (for each testing fold) was used for validation. The rest were used for training. Model hyperparameters (box constraint and lambda) were optimized using Bayesian optimization.

Models are tested using various feature subsets, namely one model per EEG feature subtype (total of five models), one per peripheral feature subtype (five total), one model for all combined EEG features, one model for all combined peripheral features, and, lastly, one model fusing both the EEG and peripheral features. Cohen's kappa is used to gauge classifier performance. Cohen's kappa is a measure that express the agreement between true class labels and models prediction (Billinger et al., 2012). This measure is commonly used in the B/BCI literature (Schlögl et al., 2005; Hasan et al., 2015). A Cohen's kappa of 0 means that the model is doing no better than chance (i.e., the accuracy would be close to 50% if classes were balanced). A Cohen's kappa of 1 means that the model is perfect (i.e., 100% accuracy).

## 5. Database Validation: Experimental results

The majority of the participants completed all six experimental conditions. Five participants decided to not perform the Outlast scenario and two participants did not fully complete TIMEframe scenarios. The most common stated cause for early interruption was nausea (possibly induced by the proximity with the screen).

Only one participant reported smoking. No participant reported suffering from hypertension. Subjective weight was reported on a four-point scale (insufficient, normal, excess, great excess). Participants reported having either a normal weight (36 participants) or an excess of weight (11 participants). One participant did not answer the weight question and none reported an insufficient weight or a great excess of weight. A majority of participants reported doing at least 30 min of exercise per day (33 participants, one did not answer). Regarding job activity levels, twenty-five participants reported having a sedentary job (e.g., office job), 13 reported having a low physical job (e.g., housekeeping, woodworking), and only three reported having a moderate physical job (e.g., construction, farming). No participant reported having a heavy-physical job (e.g., carpentry).

Moreover, in the original experimental design, all three scenes used in Outlast (i.e., Admin, Ward, Sewers) were intended to be considered as high stress conditions. It is possible, however, that some scenes were not as stressful as others. To verify this, a preliminary set of repeated measures models were fitted using only the data from Outlast session. This set used the NASA-TLX stress and fear questions as independent variable. Physical activity levels (0, 18, 24 km/h) and condition (Admin, Ward, Sewers) were used as dependant variables. Results of the repeated measure ANOVA suggest that there were no differences between all three Outlast scenes (*p*_*stress*_ > 0.05, *p*_*fear*_ > 0.05). Therefore, all Outlast scenes will be pooled under high stress in the subsequent analysis.

The following section will detail the results of the subjective, neurophysiological and peripheral measures, as well as the modeling analysis in order to validate the protocol and database.

### 5.1. Subjective Results

Table 5 reports the average scores and the mean confidence interval (confidence level of 95%) of the two subjective questionnaires across all six conditions. As can be seen, while the stress levels of the video games had an effect on the reported physical demand scores, the different physical activity levels produced no difference in the reported stress levels scores.

**Table 5:** Descriptive statistics of subjectives measures.

**Variable**	**Condition average (stress–physical activity)**					
	**Low-0 km/h**	**Low-18 km/h**	**Low-24 km/h**	**High-0 km/h**	**High-18 km/h**	**High-24 km/h**
**NASA-TLX**						
Mental demand	3.8 ± 0.8	4.1 ± 1.0	4.4 ± 0.7	11.1 ± 1.2	11.2 ± 1.1	11.6 ± 1.3
Physical demand	2.4 ± 0.9	5.4 ± 0.8	8.2 ± 1.0	3.1 ± 0.8	7.5 ± 1.1	9.8 ± 1.4
Temporal demand	3.1 ± 0.7	4.0 ± 0.8	4.4 ± 1.0	9.5 ± 1.5	10.0 ± 1.3	11.4 ± 1.4
Performance	11.2 ± 1.7	11.6 ± 1.5	11.2 ± 1.7	12.6 ± 1.7	13.1 ± 1.4	12.3 ± 1.5
Effort	6.8 ± 1.5	7.6 ± 1.3	8.6 ± 1.2	11.1 ± 1.4	11.5 ± 1.1	12.4 ± 1.2
Frustration	4.2 ± 1.3	4.2 ± 1.2	4.1 ± 1.3	9.4 ± 1.6	9.4 ± 1.6	10.0 ± 1.5
Stress^*^	2.3 ± 0.6	2.5 ± 0.6	2.4 ± 0.6	13.5 ± 1.6	13.0 ± 1.4	13.2 ± 1.4
Fear^*^	1.1 ± 0.1	1.1 ± 0.1	1.1 ± 0.1	12.2 ± 1.7	11.3 ± 1.5	12.6 ± 1.5
**BORG**						
After condition	7.0 ± 0.6	8.5 ± 0.6	9.8 ± 0.7	8.1 ± 0.6	10.0 ± 0.7	11.2 ± 0.7
After break	7.0 ± 0.4	8.0 ± 0.6	8.8 ± 0.7	8.1 ± 0.6	8.9 ± 0.7	10.1 ± 0.8

* *Stress and fear are not part of the original NASA-TLX. See Table 2*.

To better understand the effects of stress and physical activity had on subjective measures, a series of repeated measures ANOVAs are performed on NASA-TLX and BORG responses. For dimensions of the subjective rating that did not have normally distributed residuals, we performed a Friedman non-parametric test (Table 6). Stress, physical activity level and the interaction between both are used as independent variables. Greenhouse-Geisser correction of the *p* values was used when assumption of sphericity was violated. The significance level was (*p* < 0.005) after Bonferroni correction was used for multiple comparisons. Results show that the stress manipulation had an effect on most of the subjective variables (except performance) as well as on the two BORG measures. Physical activity had an effect on NASA-TLX physical demand, temporal demand, and effort, as well as on the two BORG measures. No relevant interaction was found.

**Table 6:** Results of repeated measures ANOVA for subjective measures.

**Independent variable**	**Stress**			**Physical activity**			**Stress** **×** **Physical activity**		
	**F**	**p**	**$\eta_{p}^{2}$**	**F**	**p**	**$\eta_{p}^{2}$**	**F**	**p**	**$\eta_{p}^{2}$**
**NASA-TLX**									
Mental demand^‡^	244.9	<0.001	0.85	3.6	0.031	0.08	0.3	0.722	0.00
Physical demand^†^^,^^‡^^,^^◇^	21.8	<0.001	0.35	103.4	<0.001	0.72	3.2	0.046	0.07
Temporal demand^‡^^,^^◇^	106.0	<0.001	0.72	8.6	<0.001	0.17	0.6	0.533	0.01
Performance	6.6	0.013	0.14	0.6	0.525	0.01	0.1	0.854	0.00
Effort^†^^,^^‡^^,^^◇^	66.8	<0.001	0.62	8.8	<0.001	0.18	0.2	0.767	0.00
Frustration	53.9	<0.001	0.57	0.5	0.557	0.01	0.5	0.564	0.01
Stress^*^	237.1	<0.001	0.85	0.1	0.894	0.00	0.0	0.927	0.00
Fear^*^	209.4	<0.001	0.83	2.7	0.067	0.06	2.8	0.063	0.06
**BORG**									
After condition^†^^,^^‡^^,^^◇^	36.2	<0.001	0.47	58.0	<0.001	0.59	2.3	0.101	0.05
After break^†^^,^^‡^^,^^◇^	20.2	<0.001	0.33	20.0	<0.001	0.33	0.3	0.700	0.00

* *Stress and fear are not part of the original NASA-TLX. See Table 2*.

† *Difference found for multiple comparison test (Tukey-Kramer) between 0 and 18 km/h (p < 0.05)*.

‡ *Difference found for multiple comparison test (Tukey-Kramer) between 0 and 24 km/h (p < 0.05)*.

◇ *Difference found for multiple comparison test (Tukey-Kramer) between 18 and 24 km/h (p < 0.05)*.

### 5.2. EEG Results

Table 7 reports the average values and the mean confidence interval (confidence level of 95%) of the selected EEG features across all six conditions. To analyze these results, repeated measures ANOVA is performed with the same independent variables as for Table 6. Greenhouse-Geisser correction of the *p* values was used when assumption of sphericity was violated. The significance level was (*p* < 0.0028) after Bonferroni correction was used for multiple comparisons. Table 8 reports the ANOVA results.

**Table 7:** Descriptive statistics of EEG features.

**Variable (unit)**	**Condition average (stress–physical activity)**					
	**Low-0 km/h**	**Low-18 km/h**	**Low-24 km/h**	**High-0 km/h**	**High-18 km/h**	**High-24 km/h**
**Abs. power (dB)**						
Alpha (AF7)	0.60 ± 0.38	0.68 ± 0.40	0.67 ± 0.41	0.70 ± 0.44	0.60 ± 0.38	0.52 ± 0.36
Theta (AF7)	1.14 ± 0.71	1.28 ± 0.73	1.28 ± 0.76	1.31 ± 0.82	1.09 ± 0.72	0.99 ± 0.66
Alpha (AF8)	0.87 ± 0.23	1.02 ± 0.28	0.96 ± 0.27	1.04 ± 0.27	0.88 ± 0.28	0.95 ± 0.26
Theta (AF8)	1.66 ± 0.44	1.94 ± 0.52	1.84 ± 0.50	1.97 ± 0.51	1.73 ± 0.53	1.83 ± 0.48
**Rel. power (10^−3^)**						
Gamma (AF7)	179.4 ± 32.6	178.1 ± 35.7	166.1 ± 33.6	200.4 ± 38.1	161.1 ± 37.2	163.8 ± 38.3
Gamma (AF8)	250.8 ± 38.5	228.6 ± 41.1	221.3 ± 40.6	225.5 ± 34.4	200.0 ± 37.8	222.5 ± 39.7
Gamma (TP9)	56.3 ± 20.9	40.7 ± 10.4	52.0 ± 13.1	79.9 ± 21.0	55.5 ± 13.2	56.3 ± 15.5
Gamma (TP10)	54.5 ± 11.1	38.4 ± 9.7	41.7 ± 10.1	70.0 ± 14.8	50.2 ± 12.5	52.8 ± 13.3
**Asymmetry (dB)**						
Alpha (AF7-AF8)	0.90 ± 0.45	0.90 ± 0.43	0.88 ± 0.44	0.89 ± 0.48	0.82 ± 0.43	0.90 ± 0.47
Alpha (TP9-TP10)	−0.04 ± 0.12	0.02 ± 0.11	0.04 ± 0.11	0.11 ± 0.19	0.01 ± 0.15	0.00 ± 0.15
**Coherence (–)**						
Alpha (TP9-TP10)	0.53 ± 0.06	0.53 ± 0.06	0.52 ± 0.05	0.49 ± 0.07	0.49 ± 0.06	0.48 ± 0.07
Beta (TP9-TP10)	0.34 ± 0.05	0.30 ± 0.04	0.28 ± 0.04	0.31 ± 0.06	0.27 ± 0.05	0.27 ± 0.05
Gamma (TP9-TP10)	0.30 ± 0.06	0.25 ± 0.05	0.23 ± 0.04	0.28 ± 0.06	0.23 ± 0.04	0.21 ± 0.04
Theta (TP9-TP10)	0.65 ± 0.06	0.67 ± 0.06	0.66 ± 0.06	0.61 ± 0.07	0.63 ± 0.07	0.60 ± 0.08
**AM (10^−3^)**						
Beta-delta (AF7)	83.8 ± 10.2	75.6 ± 9.1	77.1 ± 10.9	89.4 ± 10.0	80.2 ± 10.4	76.5 ± 10.4
Gamma-delta (AF7)	81.3 ± 12.2	82.7 ± 14.2	78.3 ± 12.6	91.2 ± 14.7	78.3 ± 14.6	79.1 ± 15.5
Beta-delta (AF8)	74.3 ± 6.2	66.1 ± 5.1	66.9 ± 6.0	79.7 ± 5.1	71.8 ± 5.8	73.9 ± 5.8
Gamma-delta (AF8)	98.9 ± 12.4	90.4 ± 13.4	88.2 ± 13.2	101.2 ± 10.6	82.5 ± 11.9	89.4 ± 12.9
Beta-delta (TP9)	62.4 ± 8.6	54.7 ± 11.5	56.7 ± 12.0	73.1 ± 10.6	60.9 ± 11.5	60.4 ± 11.4
Gamma-delta (TP9)	35.7 ± 9.2	27.9 ± 9.5	32.4 ± 10.5	43.7 ± 11.5	34.8 ± 10.8	34.4 ± 11.8
Beta-delta (TP10)	59.8 ± 8.2	52.1 ± 9.5	52.3 ± 10.4	71.9 ± 9.3	58.2 ± 10.6	59.0 ± 11.0
Gamma-delta (TP10)	31.5 ± 7.6	26.0 ± 8.7	27.7 ± 9.1	39.8 ± 9.3	31.6 ± 9.3	31.9 ± 9.3

**Table 8:** Results of repeated measures analysis of variance for EEG features.

**Independent variable**	**Stress**			**Physical activity**			**Stress** **×** **physical activity**		
	**F**	**p**	**$\eta_{p}^{2}$**	**F**	**p**	**$\eta_{p}^{2}$**	**F**	**p**	**$\eta_{p}^{2}$**
**Abs. power**									
Alpha (AF7)	0.4	0.533	0.01	0.9	0.409	0.02	0.8	0.442	0.02
Theta (AF7)	0.3	0.559	0.01	0.8	0.438	0.02	0.7	0.475	0.02
Alpha (AF8)	0.0	0.949	0.00	0.5	0.587	0.01	3.9	0.026	0.10
Theta (AF8)	0.0	0.961	0.00	0.2	0.762	0.01	2.9	0.059	0.08
**Rel. power**									
Gamma (AF7)	2.1	0.159	0.05	1.3	0.281	0.03	2.8	0.076	0.07
Gamma (AF8)^†^^,^^‡^	0.6	0.802	0.00	5.2	0.009	0.13	2.0	0.139	0.05
Gamma (TP9)	15.1	<0.000	0.30	2.3	0.130	0.06	7.3	0.002	0.17
Gamma (TP10)^†^	15.7	<0.001	0.30	7.0	0.004	0.16	0.7	0.476	0.02
**Asymmetry**									
Alpha (AF7-AF8)	1.14	0.293	0.03	0.8	0.452	0.02	0.02	0.846	0.00
Alpha (TP9-TP10)	0.6	0.457	0.02	0.2	0.762	0.00	1.0	0.356	0.03
**Coherence**									
Alpha (TP9-TP10)	4.7	0.037	0.12	0.1	0.932	0.00	0.2	0.844	0.00
Beta (TP9-TP10)^†^	2.1	0.159	0.05	5.0	0.018	0.12	0.5	0.951	0.00
Gamma (TP9-TP10)^†^^,^^‡^	1.4	0.247	0.04	10.0	<0.001	0.22	0.9	0.409	0.02
Theta (TP9-TP10)	4.9	0.032	0.12	0.1	0.909	0.00	0.7	0.488	0.02
**AM**									
Beta-delta (AF7)	2.9	0.099	0.07	2.6	0.096	0.07	2.7	0.082	0.07
Gamma-delta (AF7)	4.1	0.051	0.10	0.6	0.518	0.02	3.1	0.061	0.08
Beta-delta (AF8)^†^^,^^‡^	6.8	0.014	0.16	8.0	<0.001	0.18	0.1	0.943	0.00
Gamma-delta (AF8)^†^^,^^‡^	0.5	0.482	0.01	5.6	0.008	0.13	1.9	0.149	0.05
Beta-delta (TP9)	6.9	0.012	0.16	2.1	0.097	0.07	1.5	0.222	0.04
Gamma-delta (TP9)	13.8	<0.001	0.28	1.7	0.196	0.05	5.3	0.008	0.13
Beta-delta (TP10)^†^	8.9	0.005	0.20	3.6	0.043	0.09	1.4	0.262	0.04
Gamma-delta (TP10)^†^	14.3	<0.001	0.28	4.4	0.019	0.11	2.1	0.140	0.05

† *Difference found for multiple comparison test (Tukey-Kramer) between 0 and 18 km/h (p < 0.05)*.

‡ *Difference found for multiple comparison test (Tukey-Kramer) between 0 and 24 km/h (p < 0.05)*.

As can be seen, prefrontal absolute power of alpha and theta was not significantly altered by the experimental manipulations. The ANOVA did not reveal any effects on relative prefrontal gamma. However, results suggest that relative gamma in temporal-parietal regions was higher during the stress condition (TP9, *p* < 0.001, $\eta_{p}^{2}=0.30$; TP10, *p* < 0.001, $\eta_{p}^{2}=0.30$), although they were not affected by physical activity, but an interaction was present at this location (*p* = 0.002, $\eta_{p}^{2}=0.17$). The asymmetry and coherence features did not reveal any effect or interaction. Amplitude modulation features showed sensitivity to the stress manipulation. This sensitivity appeared higher for gamma modulated by delta for temporal-parietal regions (TP9, *p* < 0.001, $\eta_{p}^{2}=0.28$; TP10, *p* < 0.001, $\eta_{p}^{2}=0.28$). Globally, amplitude modulation was elevated by stress and lowered by physical activity.

### 5.3. Peripheral Results

Table 9 reports the average values and the mean confidence interval (confidence level of 95%) of the selected peripheral features across all six conditions. As previously, repeated measures ANOVA is performed with Greenhouse-Geisser correction of the *p* values was used when assumption of sphericity was violated. The significance level was (*p* < 0.0036) after Bonferroni correction was used for multiple comparisons. Table 10 reports the ANOVA results.

**Table 9:** Descriptive statistics of peripheral measures.

**Variable (unit)**	**Condition average (stress–physical activity)**					
	**Low-0 km/h**	**Low-18 km/h**	**Low-24 km/h**	**High-0 km/h**	**High-18 km/h**	**High-24 km/h**
**Cardiac**						
Heart rate (*bpm*)	85.0 ± 3.9	91.5 ± 4.0	94.6 ± 4.2	86.7 ± 4.1	92.9 ± 4.0	95.5 ± 4.1
SDNN (*ms*)	46.5 ± 5.6	37.3 ± 4.5	36.3 ± 5.0	53.8 ± 6.3	42.0 ± 4.6	41.0 ± 5.9
HF power (*ms*^2^)	7.1 ± 1.8	9.1 ± 2.7	9.0 ± 2.2	6.1 ± 1.7	6.4 ± 1.5	8.1 ± 2.6
LF/HF (–)	4.3 ± 0.7	3.9 ± 0.8	3.7 ± 0.8	4.4 ± 1.1	4.7 ± 1.1	4.3 ± 1.1
**Breathing**						
Breathing rate (*breath*/*min*)	22.4 ± 1.5	26.0 ± 1.6	24.9 ± 1.1	23.4 ± 1.4	27.0 ± 1.5	25.7 ± 1.4
Variability (–)	0.71 ± 0.10	0.83 ± 0.09	0.74 ± 0.10	0.71 ± 0.09	0.81 ± 0.10	0.73 ± 0.11
Sigh rate (*min*^−1^)	2.36 ± 0.25	3.26 ± 0.38	2.88 ± 0.25	2.23 ± 0.23	2.84 ± 0.34	2.60 ± 0.27
**EDA**						
Level (–)	0.96 ± 0.22	1.06 ± 0.39	1.29 ± 0.37	1.07 ± 0.24	1.24 ± 0.30	1.10 ± 0.21
Number of peaks (*n*)	46.6 ± 3.0	49.2 ± 2.4	48.8 ± 1.8	47.3 ± 2.4	50.7 ± 1.7	50.6 ± 1.3
Rel. LF power (–)	0.10 ± 0.02	0.15 ± 0.03	0.12 ± 0.03	0.13 ± 0.03	0.16 ± 0.03	0.16 ± 0.03
**Skin temperature**						
Temperature (°C)	33.8 ± 0.6	33.6 ± 0.5	33.6 ± 0.4	33.6 ± 0.6	33.4 ± 0.5	33.4 ± 0.5
Temperature delta (°C)	0.34 ± 0.11	0.24 ± 0.11	0.22 ± 0.12	0.13 ± 0.10	0.12 ± 0.13	0.12 ± 0.10
**Blood volume pulse**						
Minimum BVP (–)	0.68 ± 0.13	0.78 ± 0.12	0.82 ± 0.17	0.91 ± 0.19	0.95 ± 0.15	1.01 ± 0.24
Maximum BVP (–)	0.72 ± 0.14	0.86 ± 0.14	0.91 ± 0.17	0.94 ± 0.19	0.88 ± 0.13	1.07 ± 0.22

**Table 10:** Results of repeated measures analysis of variance for peripheral measures.

**Independent variable**	**Stress**			**Physical activity**			**Stress** **×** **Physical activity**		
	**F**	**p**	**$\eta_{p}^{2}$**	**F**	**p**	**$\eta_{p}^{2}$**	**F**	**p**	**$\eta_{p}^{2}$**
**Cardiac**									
Heart rate^†^^,^^‡^	2.7	0.109	0.07	10.5	<0.001	0.23	0.2	0.831	0.00
SDNN^†^^,^^‡^	10.8	0.002	0.23	8.2	<0.001	0.19	0.2	0.837	0.00
HF power	2.0	0.172	0.06	2.1	0.133	0.06	1.2	0.308	0.04
LF/HF	1.7	0.198	0.05	0.3	0.693	0.01	0.7	0.470	0.02
**Breathing**									
Breathing rate^†^	12.9	<0.001	0.26	5.6	0.007	0.13	0.3	0.727	0.01
Breathing variability	0.3	0.589	0.01	1.4	0.262	0.04	0.3	0.704	0.01
Sigh rate^†^	6.0	0.019	0.14	7.2	0.002	0.17	0.5	0.587	0.01
EDA									
EDA Level	0.4	0.522	0.01	0.6	0.479	0.02	2.2	0.130	0.06
EDA responses	3.3	0.077	0.09	2.9	0.071	0.08	0.4	0.639	0.01
EDA Rel. LF power	3.4	0.074	0.09	2.5	0.091	0.07	0.2	0.828	0.01
**Skin temperature**									
Temperature	3.2	0.082	0.08	1.0	0.359	0.03	0.2	0.817	0.00
Temperature delta	10.4	0.003	0.23	0.7	0.479	0.02	0.7	0.504	0.02
**Blood volume pulse**									
Minimum BVP	14.2	<0.001	0.30	0.9	0.402	0.03	0.5	0.568	0.02
Maximum BVP	9.9	0.003	0.23	1.6	0.222	0.04	1.5	0.238	0.04

† *Difference found for multiple comparison test (Tukey-Kramer) between 0 and 18 km/h (p < 0.05)*.

‡ *Difference found for multiple comparison test (Tukey-Kramer) between 0 and 24 km/h (p < 0.05)*.

For the cardiac features, ANOVA suggests that heart rate rose as physical activity was more intense ($p<0.001,\eta_{p}^{2}=0.23$). The SDNN was also significantly higher during high stress ($p=0.002,\eta_{p}^{2}=0.23$) and decreased with more intense physical activity ($p<0.001,\eta_{p}^{2}=0.19$). No effects or interactions were detected for spectral features of heart rate variability (HR, LF/HF ratio). Breathing rate was higher in the stress condition ($p<0.001,\eta_{p}^{2}=0.26$) and higher during physical activity, although it was not affect by physical activity. ANOVA revealed an effect of physical activity on sigh rate ($p=0.002,\eta_{p}^{2}=0.17$). Table 9 suggests that sigh rate was higher in presence of physical activity, but slightly higher for the 18 km/h level of physical activity. No effects or interaction was detected for breathing variability. Electrodermal features did not reveal any effect of experimental conditions, although some positive trends could be observed (e.g., number of responses, *p*_*stress*_ = 0.077, *p*_*physical*_ = 0.071; Rel. LF power, *p*_*stress*_ = 0.074, *p*_*physical*_ = 0.091). Temperature also did not appear to vary across the two experimental manipulations. However, results suggest that the temperature delta was significantly affected by the stress manipulation ($p=0.003,\eta_{p}^{2}=0.23$). More specifically, temperature delta was much lower during high stress conditions. ANOVA also suggested that minimum and maximum BVP were much higher during high stress conditions (minimum, $p<0.001,\eta_{p}^{2}=0.30$; maximum, $p=0.003,\eta_{p}^{2}=0.23$). No effect of physical activity or interaction was found for temperature or BVP features.

### 5.4. Modeling Results

**Figure 5:**
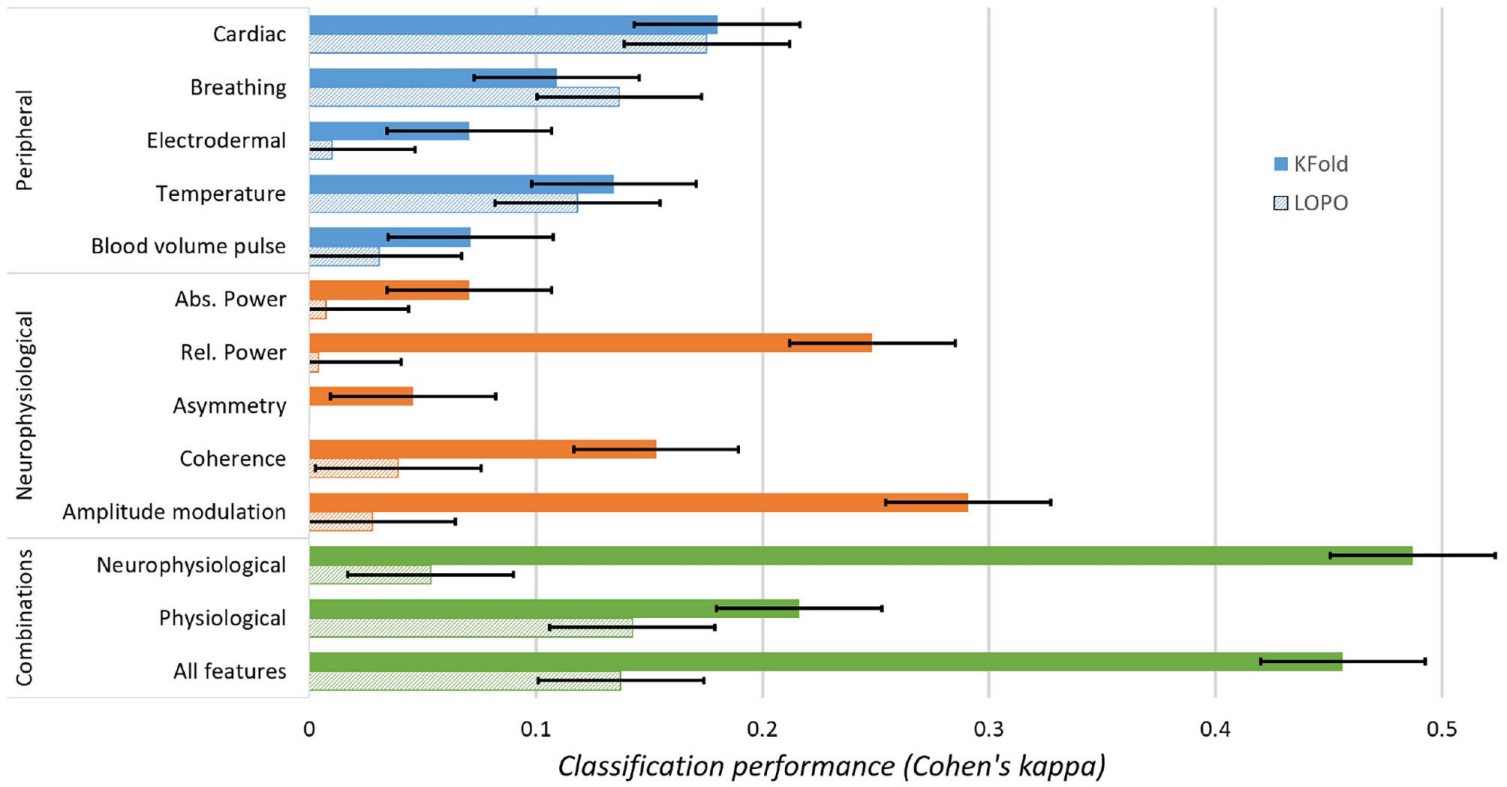
Classification performance (Cohen's kappa) of different feature subsets.

A two-way ANOVA is performed using feature subsets and testing schemes as dependent variables. Results from the ANOVA suggest the presence of a significant difference between at least two features subsets (*p* < 0.001), between the two schemes (*p* < 0.001) and an interaction between both factors (*p* < 0.001). To further understand these results, a multiple comparison analysis is performed and Tukey's honest significant difference is used to correct multiple comparisons. Figure 5 shows the results of these multiple comparisons. For k-fold, the best classification performance was obtained using either all features (κ_*avg*_ = 0.46, *acc* = 0.73%) or all EEG features (κ_*avg*_ = 0.49, *acc* = 0.74%). Amplitude modulation features were the best single type of feature type (κ_*avg*_ = 0.29, *acc* = 0.65%), significantly surpassing the combination of all peripheral features (κ_*avg*_ = 0.22, *acc* = 0.61%), as well as all other single type of features (except relative power features, κ_*avg*_ = 0.25, *acc* = 0.63%). Cardiac features yielded the best performance for single peripheral feature type (κ_*avg*_ = 0.18, *acc* = 0.60%), surpassing EDA, BVP, absolute power and asymmetry features (which all yielded relatively poor results, κ_*avg*_ < 0.10, *acc*_*avg*_ = 0.53%). Peripheral features provided the most stable results across the two testing schemes. EEG features, on the other hand, all performed very poorly under the LOPO scheme, suggesting that subject-specific models are needed, or more advanced normalization strategies (Albuquerque et al., 2019a).

## 6. Discussion

As stated in the introduction, this project features two main goals. First, we want to provide a dataset where stress and physical activity are jointly modulated. We also seek to allow exploration of physical activity on artifact generation. Second, we want to provide a dataset that mimics realistic settings to support “in-the-wild” B/BCI development. In the following section, we provide a discussion of the analysis that were performed in order to better characterize the dataset.

### 6.1. Subjective Analysis

The important effect of the stress manipulation on the custom stress and fear questions suggest that the experimental manipulation was successful. The stress manipulation also had an important effect on mental demand. This result might have been caused by the games design. Despite being similar in terms of game style (first person exploration games), Outlast featured more complex environmental design (e.g., dead ends, hidden passages) than TIMEframe. Outlast also had more complex controls (e.g., using the night mode, running), which might also explain the increased perceived physical demand. Despite knowing that the two games had a predetermined duration (10 min), participants felt that Outlast caused higher temporal demand than TIMEframe. This result might be due to the escape scenes (i.e., escaping from chasing enemies) that were present in all three Outlast scenarios. Overall, it is clear that the stress manipulation caused a high affective stress state, as well as induced some mental stress. This highlights the difficulty in experimentally separating the two forms of stress, a limitation shared with other popular protocols, such as the Trier Social Stress Task (Kudielka et al., 2007).

The physical activity manipulation was also successful, this can be appreciated even with the *p* values corrected for multiple comparisons between the three level. The descriptive statistics and the straightforward difference between physical activity levels (0, 18, and 24 km/h) suggest that the participants did feel more physical demand as activity levels increased. Results suggest that participants felt a slightly higher temporal demand as physical activity rose. It is important to keep in mind that the higher speed, like any physical activity manipulation, might have induced a higher mental demand on participants. This might have translated into higher subjective temporal demand. Moreover, the effect of physical activity on the effort rating was expected to be higher. However, is it possible that some participants considered this question to concern mental effort, while others physical effort, thus canceling out any potential effects.

### 6.2. EEG Analysis

Absolute power of alpha and theta did not vary significantly under stress. Since these features were only computed in prefrontal regions, it is possible that they were strongly affected by ocular artifacts. In fact, all prefrontal features computed did not reveal much sensitivity to stress. It could be argued that the task visual load was too high to fully remove all artifacts, suggesting that prefrontal sensitivity to stress could be higher when eyes are closed compared to when eyes are open, as it was reported in Brouwer et al. (2011).

The difference between mental and affective stressors might also explain the absence of effects on prefrontal alpha and theta. In Borghini et al. (2014), the authors mention that the expected decrease of alpha and increase of theta are observed in situation where the task demand is higher. In Giannakakis et al. (2015), authors report several significant differences on absolute power of alpha, beta and theta bands using a more affective than mental stressor (i.e., video segments). Like in Borghini et al. (2014), they do observe lower alpha power in frontal regions (i.e., F3). However, they did not report differences in the locations used in this study. Following this hypothesis, it is also possible that the two video games induced similar mental stress on participants, making it difficult to observe a difference. Additionally, it is also possible that physical activity reduced the experienced stress, thus making it more difficult to be detected. Finally, physical activity might have induced movement on the headset, leading to a poorer contact between the electrodes and the skin. This indeed is a limitation of using the Muse headband.

Notwithstanding, stress had a very clear effect on temporal-parietal relative gamma (on both sides). These results are in line with the hypothesis (see Table 3) that relative gamma would rise under stress as per (Minguillon et al., 2016), where the authors focus more on the role of prefrontal relative gamma (rather than temporal-parietal, like in the present study). However, they do report that relative gamma also increased in temporal and parietal regions. In Minguillon et al. (2016), it is suggested that prefrontal relative gamma could be an indicator of mental stress, rather than affective stress (a result supported by previous studies, Başar-Eroglu et al., 1996). In another work, temporal and parietal gamma were found to be higher in presence of an affective stressor (Oathes et al., 2008). Given that the current study focused more on affective stress, it is possible that participants experienced similar mental stress in the two games played; this hypothesis is based on the stress level effect on the temporal relative gamma, and the no effect of prefrontal relative gamma. As suggested before, it is possible that ocular and physical activity artifacts seen here prevented detecting a stress effect on prefrontal relative gamma. Moreover, the removal of those artifacts with wICA could have negatively impacted the high frequency components in EEG signals in the prefrontal region (Muthukumaraswamy, 2013; Cassani et al., 2014; Rosanne et al., 2019). Together, these results suggest that stress “in-general” might be associated with the gamma band and that the prefrontal/temporal-parietal predominance might indicate whether this stress is more mental or affective. Further work would be required to confirm this.

Under stress, interhemispheric temporal-parietal coherence (TP9-TP10) was slightly lower, suggesting a less similar neuronal activity between the two regions. This result goes against the hypothesis formulated in Table 3 (Giannakakis et al., 2015). In Giannakakis et al. (2015), authors found that alpha coherence was higher during stressful video segments compared to relaxed segments (although in parietal region, P3-P4). However, coherence behavior under stress is not well-documented in the literature. In Travis et al. (2010), parietal interhemispheric alpha1 (7.5–10.0 Hz) coherence was higher during meditation compared to control. While we cannot directly compare the TIMEframe game to meditation, it could be argued that the relaxed states enhance interhemispheric coherence.

Lastly, amplitude modulation features yielded several interesting results. Globally, amplitude modulation rose during stress conditions. The apparent larger increase observed on temporal-parietal regions might, once again, have been caused by the hypothesized greater influence of ocular and physical activity artifacts on prefrontal electrodes. Consistent with relative gamma effects, amplitude modulation effects were also greater when observed in the gamma band. It could be argued that the high amplitude modulation observed on gamma (TP9-TP10) are due to fluctuation in experienced stress during the Outlast play session (as opposed to TIMEframe, which induced had a lower and more leveled stress level). In this paper, we explored only a subset of possible amplitude modulation features (i.e., delta-modulated) and future work should explore alternate features. For mental workload assessment, for example, they also showed to be important (Albuquerque et al., 2019b).

### 6.3. Peripheral Analysis

The increased heart rate observed with physical activity confirms that the physical activity manipulation was effective. As expected, SDNN also rose during high stress conditions (Castaldo et al., 2015). This result reinforces the utility of SDNN as an index of affective stress. However, the observed decrease of SDNN under higher physical activity levels suggest that this feature could have higher predictive power if physical activity of individuals was unknown. Despite being shown sensible to stress in other studies (Kreibig et al., 2007), spectral features of heart rate variability (i.e., HF power, LF/HF ratio) were not significantly affected by the stress manipulations. Since physical activity is known to change heart rate variability, it is possible that physical activity acted as a confounding factor (Pichon et al., 2004). For example, in Pichon et al. (2004), the LF/HF ratio is reported to decrease as physical activity rises. This behavior might have canceled the expected increase that was hypothesized in Table 3. It is also possible that the relatively fast changes in physical activity intensity prevented these features from reaching temporal stability. In addition, the LF/HF ratio has received some criticism as a measure of cognitive and physical aspects of stress, as its correspondence to psychological and physiological states of a person is not unique, and by combining LF and HF one degree of freedom is lost. Future studies could explore the effects on LF and HF separately (von Rosenberg et al., 2017) or investigate potentially more relevant features for ambulant users (e.g., Tiwari et al., 2019, 2020). Finally, it is also possible that spectral features of heart rate variability are more associated with mental stressors than affective stressors. The hypothesis made in Table 3 are based on Castaldo et al. (2015), which predominantly features mental stressors.

As expected, breathing rate rose under higher physical activity conditions. In concordance with our hypothesis, stress increased the breathing rate (Rainville et al., 2006). The effect size of stress on breathing rate was higher than from physical activity. Surprisingly, sigh rate was lower during stressful conditions, which is opposed to our formulated hypothesis (Vlemincx et al., 2013). Participants might have sighed only once the threat was removed (i.e., after the condition). Given that Outlast's played character is often chased and threatened, it is also possible that participants unconsciously held their breath as not to make noise. The absence of effect on breathing variability might have been caused by the parameters used to compute sample entropy (*m*, and *r*). In Vlemincx et al. (2013), authors mentioned that they used *m* = 2 and *r* = 0.4 and these were the parameters used herein. However, it is uncertain if these parameters are optimal for all situations.

The absence of significant effects of stress and physical activity on all electrodermal features was counter-intuitive, as both stress and physical activity have been shown to induce changes in EDA patterns. Placement of the electrodes might partially explain the lack of concordance with the literature. In stress related experiments, electrodes are often placed on the fingers (e.g., Kreibig et al., 2007; Posada-Quintero et al., 2016b, 2018a) or on the foot (e.g., Reinhardt et al., 2012). In setups involving physical activity, it can be more practical to use a wristband (e.g., Gjoreski et al., 2016) as was the case with the current study). It is also possible that the combined affective stressor and physical activity saturated the EDA levels, thus creating a ceiling effect and preventing variability. In Posada-Quintero et al. (2018b), physical activity was manipulated while EDA was recorded. While authors did observe significant difference between the different physical activity levels, they mention that the electrodermal level and the number of responses did not have the sensitivity of spectral features. Physical activity might have also introduced artifacts to the electrodermal measure. Precautions were taken to prevent this: the wrist band was sufficiently tightened to prevent slippage and filtering was applied to the signal to remove higher frequency noise. Finally, it is possible that alternate frequency bands could have achieved improved discriminability. As spectral analysis of EDA is still a relatively undocumented domain, further improvements may be possible.

Lastly, the hypothesis that temperature would be lower during high stress condition (see Table 4) was not confirmed. This could be due to the counter-effect of physical activity, which is known to increase body temperature (Lim et al., 2008) even in areas not directly involved in the effort (Chudecka and Lubkowska, 2012). In line with the hypothesis, however, temperature rose much more slowly during stress conditions. As stated in section 2, this could be due to a constriction of the limb arterioles, intended to reduce blood flow in peripheral regions during fight-or-flight situations. This observation matches the increase in blood volume pulse that was induced during stress conditions (Kreibig et al., 2007). It is interesting to note that none of the temperature and blood volume pulse measures were significantly affected by physical activity. Given the short duration of the experimental conditions and the relatively low intensity of the physical task, this behavior is likely not to generalize to all forms of physical activity.

### 6.4. Modeling Analysis

The goal of the modeling analysis described here was to perform a first validation of the discriminative power of neurophysiological features for stress monitoring under physical activity, and not necessarily to obtain state-of-the-art results (Smets et al., 2019). As such, default classifier parameters were used and classical SVMs were tested. Our ongoing study involves the use of multimodal fusion and classifier optimization to further improve results. The interested reader is referred to Parent et al. (2019b) for more details.

Interestingly, while both EEG and peripheral feature subsets showed similar effect size under stress ($\eta_{2}^{p}\approx 0.25$), classification performance differed largely between them. For example, while peripheral features resulted in lower stress prediction performance under the k-fold setting relative to EEG, they generalized better to unseen users in the LOPO scheme. This sensitivity has been reported previously for EEG-based mental workload models (Albuquerque et al., 2019a).

Combining all EEG feature subsets also significantly improved classification results, thus corroborating results reported in the mental workload literature (Albuquerque et al., 2019b). On the other hand, combining EEG with peripheral features did not result in performance gains. Peripheral measures, such as heart rate variability and/or electrodermal activity are often viewed as generic indicators of sympathetic and parasympathetic activation (Billman, 2011; Posada-Quintero et al., 2016a), thus they may provide limited concurrent sources of information, especially in the presence of physical activity.

Moreover, as stated previously, the amplitude modulation features were shown to result in the highest performance under the k-fold setting. Here, only a subset of possible AM features was computed and recent work has suggested that alternate bands can be useful for valence and arousal prediction (Clerico et al., 2018). Future work will explore the full potential of the amplitude modulation features for stress prediction under physical activity.

Within the peripheral modality, cardiac features resulted in the best performance under both testing paradigms. Here, only four cardiac features were explored and relied on time- and frequency-based content. There have been recent innovations in HRV analysis showing that non-linear features may provide improved robustness to noisy data (Tobon et al., 2017), thus improved performance may still be achieved; this is left for future work.

Overall, the modeling analysis results presented herein confirm that affective stressors can induce detectable effects on neuro-physiological signals, despite being in the presence of quickly shifting physical activity. It is hoped that the database provided will allow for other researchers to help advance the knowledge of physiological stress monitoring in the presence of physical activity. This could have important implications for operator functional state monitoring for e.g., first responders.

Lastly, we performed a sanity check to explore the intensity of confound between stress and physical activity. To this end, we performed feature ranking using the recursive feature elimination algorithm. We first found the most important features for stress level detection and trained a classifier on these features to classify physical activity level; we found a Cohen's kappa value of 0.14. In turn, we found the best features for physical activity level classification and used those features to classify stress level; we found a Cohen's kappa of 0.07. Future work could explore the use of physical activity-level aware classification for improved accuracy, as in Sun et al. (2010).

### 6.5. Future Research Directions

We believe the PASS dataset analysis unlocked many questions and challenges that can be further addressed and investigated by future work. In the following, we summarize some of the research avenues that can be derived from our proposed dataset:

- Design analyses that aim to disentangle the effects of affective and mental stress components on subjective, neurophysiological, and peripheral measures (e.g., evaluate whether different modalities are affected by affective and mental stress in distinct ways);
- Devise EEG artifact removal approaches for data acquired with low-density devices which are also suitable to remove noise generated by physical activity;
- Assess the effect of different EEG referencing approaches on stress detection;
- Explore new features, including (but not limited to), EEG amplitude modulation features that have been linked to mental workload (Albuquerque et al., 2018) or new movement-robust heart rate variability features (Tiwari et al., 2020);
- Develop representation learning pipelines tailored to improve robustness to movement artifacts and inter-subject variability;
- Account for the interplay between stress levels and physical activity by devising stress classification strategies which are conditioned on the current physical activity intensity;
- Explore different state-of-the-art classification schemes and hyperparameter tuning strategies.

## 7. Conclusions

The dataset described herein was designed to support the development of physiological stress monitoring models for ambulant users. Two different videogames were used as stress modulators under three physical activity conditions. Our validation results suggest that accurate disambiguation between affective and mental stress effects could be observed even under varying physical activity levels. Validation experiments show features derived from the database to not only corroborate results previously reported in the literature, but to also provide new insights on stress elicitation under physical activity. Lastly, preliminary classification results with popular features and classical classifiers show the promise of stress monitoring of ambulant users with the use of off-the-shelf wearable devices. The collected database, comprised of raw signals, subjective ratings, and triggers, is available for download at http://musaelab.ca/pass-database/.

## Data Availability Statement

The datasets generated for this study are available on request to the corresponding author.

## Ethics Statement

Written informed consent was obtained from the individuals for the publication of any potentially identifiable images or data included in this article.

## Author Contributions

All authors: experimental design and writing and reviewing. MP, IA, AT, and RC: statistical analysis and programming. ST and TF: funding and supervision.

## Conflict of Interest

J-FG and DL were employed by the company Thales Research and Technology Canada. The remaining authors declare that the research was conducted in the absence of any commercial or financial relationships that could be construed as a potential conflict of interest.
